# Fluoroquinolone Resistance in *Campylobacter jejuni* Isolates in Travelers Returning to Finland: Association of Ciprofloxacin Resistance to Travel Destination

**DOI:** 10.3201/eid0902.020227

**Published:** 2003-02

**Authors:** Antti Hakanen, Hannele Jousimies-Somer, Anja Siitonen, Pentti Huovinen, Pirkko Kotilainen

**Affiliations:** * National Public Health Institute, Turku, Finland; † Turku University Central Hospital, Turku, Finland; ‡ National Public Health Institute, Helsinki, Finland; § Mehiläinen Hospital, Helsinki, Finland; 1 Dr. Jousimies-Somer is deceased.

**Keywords:** *Campylobacter jejuni*, quinolone, fluoroquinolone, ciprofloxacin, antibiotic,antimicrobial, drug resistance, microbial, traveler

## Abstract

Ciprofloxacin resistance was analyzed in 354 *Campylobacter jejuni* isolates collected during two study periods (1995–1997 and 1998–2000) from travelers returning to Finland. The increase in resistance between the two periods was significant among all isolates (40% vs. 60%; p<0.01), as well as among those from Asia alone (45% vs. 72%; p<0.01).

*Campylobacter jejuni* isolates are naturally susceptible to fluoroquinolones (*1*,*2*). During the 1990s, however, fluoroquinolone resistance in *Campylobacter* rapidly increased in several countries (*3*). In Thailand and Spain, for example, up to 80% of *Campylobacter* isolates are fluoroquinolone-resistant (*4*,*5*). However, major differences in *Campylobacter* fluoroquinolone resistance levels are known to occur, and in many parts of the world fluoroquinolone resistance levels remain low (*3*). This study was performed to evaluate the level of fluoroquinolone resistance in *C. jejuni* isolates from travelers returning to Finland and to specify the countries where resistant isolates are acquired.

## The Study

Our study included 354 clinical human fecal *C. jejuni* isolates collected from travelers returning to Finland from 1995 to 2000. The isolates were collected in two different phases from the laboratory of a large private hospital in Helsinki, Finland. Participants were treated as outpatients, and no data on antimicrobial usage before fecal sampling were available; all participants had a history of traveling abroad within the preceding 2 weeks. From January 1995 to November 1997, we consecutively collected 205 isolates, and from October 1998 to January 2000, 149 isolates. The isolates were cultured and identified by standard microbiologic methods (*6*). MICs of ciprofloxacin and nalidixic acid for the isolates were determined by the agar plate dilution method, as described (*7*). *C. jejuni* RH 3583 (a local control strain, originally isolated in Edinburgh, U.K., *C. jejuni* 143483) was used as a control in susceptibility testing and also as a growth control strain. The MIC breakpoint used for the resistance to ciprofloxacin was that recommended by the National Committee for Clinical Laboratory Standards (NCCLS) for non-*Enterobacteriaceae* (*8*). To nalidixic acid, the breakpoints were those recommended by NCCLS for *Enterobacteriaceae* (*8*).

Data concerning the numbers of travels from Finland to countries of interest (i.e., countries with the largest numbers of all *C. jejuni* isolates or of ciprofloxacin-resistant isolates) during the study months were received from Statistics Finland (available from: URL: www.stat.fi/). The susceptibility data were analyzed by using the WHONET5 computer program (available from: URL: www.who.int/emc/WHONET/WHONET.html).

Statistical analysis was made by using the chi-square test and the Fisher exact test. Differences between *C. jejuni* infection rates in travelers returning from various travel destinations were statistically tested with Poisson regression analysis. Differences were quantified with infection rates and 95% confidence intervals; p values of <0.05 were considered significant. Statistical data were analyzed by using the SAS system for Windows, release 8.2/2001 (SAS Institute, Inc., Cary, NC).

Of the 354 *C. jejuni* isolates studied, the country where campylobacteriosis was acquired could be identified for 319 isolates, collected from travelers to 40 different countries. The origin of 22 isolates was traced at least to a continental level; the patients involved had several travel destinations. The origin of 13 isolates remained unknown. The most common countries of origin were Spain with 77 (22%) isolates, Thailand with 50 (14%) isolates, and India with 23 (6%) isolates. During the first study period (1995–1997), 205 isolates were collected from travelers. Of the 34 countries of origin that were identified, the most common were Spain with 40 (20%) isolates, India with 19 (9%) isolates, and Thailand and Turkey, both with 17 (8%) isolates. During the second study period (1998–2000), 149 isolates were collected from travelers; of the 25 countries of origin identified, the most common were Spain with 37 (25%) isolates, Thailand with 33 (22%) isolates, and Portugal and Tunisia, both with 6 (4%) isolates.

Of all 354 *C. jejuni* isolates, 172 (49%) were resistant to ciprofloxacin. Of the 205 isolates collected in 1995–1997, 82 (40%) were resistant to ciprofloxacin compared with 90 (60%) of the 149 isolates collected in 1998–2000 (p<0.01). When analyzed by continent, the increase in fluoroquinolone resistance between these two periods was significant among the isolates from Asia alone (45% vs. 72%, p<0.01; Table 1). An increasing tendency for fluoroquinolone resistance was also observed in the isolates from the three additional continents. The numbers and proportions of ciprofloxacin-resistant isolates collected from travelers returning from Spain and Thailand, the two most common countries of origin, were analyzed separately. During the first and second collection periods, the respective numbers and proportions of ciprofloxacin-resistant isolates were 29 (73%) and 26 (70%) in the isolates from Spain, and 13 (77%) and 26 (79%) in the isolates from Thailand. To assess whether the larger proportion of isolates from Thailand during the second period (8% vs. 22%) explained the significant increase in fluoroquinolone resistance in the whole study group, we analyzed the data excluding all isolates from Thailand. The increase in ciprofloxacin resistance, from 37% to 55%, was still significant (p<0.01). A corresponding analysis that excluded all isolates from both Spain and Thailand resulted in an increase in ciprofloxacin resistance from 27% to 48% (p<0.01).

**Table 1:** Origin of 354 *Campylobacter jejuni* isolates from travelers returning to Finland and proportion of ciprofloxacin-resistant isolates during two collection periods

Geographic area	1995–1997		1998–2000	
	All isolates	% Ciprofloxacin resistant	All isolates	% Ciprofloxacin resistant
Africa	24	17	8	38
America	4	0	3	67
Asia	78	45^a^	57	72^a^
Southeast Asia	28	61	39	77
Other areas	50	36	18	61
Europe	93	44	73	51
Mediterranean area	64	59	48	69
Other areas	29	10	25	16
Total	205^b^	40^a^	149^c^	60^a^

^a^Difference is statistically significant (p<0.01).

^b^One isolate from Australia and five isolates of unknown origin included.

^c^Eight isolates of unknown origin included.

The number of ciprofloxacin-resistant *C. jejuni* isolates collected was compared with the estimated numbers of all trips from Finland during the study period to the five most frequent countries of origin for the ciprofloxacin-resistant isolates. These speculative infection rates were used to calculate rate ratios between these countries. Because the speculative infection rate by fluoroquinolone-resistant *C. jejuni* isolates was highest in travelers returning from Thailand, that country was used as the reference in the rate ratio comparisons. The rate ratios by fluoroquinolone-resistant isolates in travelers returning from Spain and Portugal were 0.11 in both groups; the differences were statistically significant compared with the ratios for the reference country (p<0.01 for both; Table 2). The corresponding rate ratios in travelers returning from India and China were 0.90 and 0.72, respectively; these differences were not significant.

**Table 2:** Rate ratios of travel-associated infections with ciprofloxacin-resistant *Campylobacter jejuni* isolate*s^a^*

Country	Estimated no. of trips from Finland during the study months^b^	No. of all isolates	Speculative infection rate^c^	Rate ratio^d^	No. of ciprofloxacin-resistant isolates	Speculative infection rate^c^ by ciprofloxacin-resistant isolates	Rate ratio^d^ by ciprofloxacin-resistant isolates
Spain (incl. Canary Islands)	1,145,872	77	(0.1)	0.12 (0.08 to 0.17)	55	(0.05)	0.11 (0.07 to 0.16)^d^
Thailand	87,842	50	(0.6)	1	39	(0.44)	1^e^
India	27,591	23	(0.8)	1.46 (0.89 to 2.40)	11	(0.40)	0.90 (0.46 to 1.75)
China	25,073	12	(0.5)	0.84 (0.45 to 1.58)	8	(0.32)	0.72 (0.34 to 1.54)
Portugal	148,647	11	(0.1)	0.13 (0.07 to 0.25)	7	(0.05)	0.11 (0.05 to 0.24)^e^

^a^The five most frequent countries of origin of ciprofloxacin-resistant *C. jejuni* isolates were included in this analysis.

^b^Based on the numbers of Finnish travelers to these countries; data collected from the reports of Statistics Finland.

^c^Infections per 1,000 trips. Since the total number of isolates from Finnish population was not studied, the rate is speculative but could be used to calculate comparable rate ratios.

^d^Thailand as the reference country; 95% confidence intervals in parentheses.

^e^Differences between Thailand and Spain, and Thailand and Portugal, are statistically significant (p<0.01).

## Conclusions

We have shown that ciprofloxacin resistance significantly increased (from 40% to 60%; p<0.01) during the study period among all *C. jejuni* isolates from travelers. The increase was also significant in isolates from Asia alone, suggesting a continual presence of selection pressure for the emergence of fluoroquinolone resistance on that continent. Moreover, an increasing tendency in ciprofloxacin resistance was observed in isolates from three additional continents, but either the increase (from 44% to 51% in Europe) or the number of isolates (7 isolates from America and 32 isolates from Africa) was small, and the changes were not statistically significant. Throughout the study, the rates of ciprofloxacin resistance remained on a high level in Spain and Thailand, the two most frequent countries of origin for all foreign isolates, as well as for resistant isolates. The fact that the increase in ciprofloxacin resistance remained significant even after all isolates from Spain and Thailand were excluded from the analysis illustrates that the emergence and spread of fluoroquinolone-resistant *C. jejuni* are not restricted to a few highly *Campylobacter*-endemic countries. Rather, these findings show that *C. jejuni* fluoroquinolone resistance, which manifested at the beginning of the 1990s, continues to grow rapidly in many parts of the world.

Several studies have focused on the quinolone resistance of *Campylobacter* spp. In Spain, a rapid increase in quinolone resistance was observed after 1988, with up to 50% of *C. jejuni* isolates resistant by 1991 (*9*,*10*). In recent years, fluoroquinolone resistance rates among Spanish *C. jejuni* isolates have been reported to reach 80% (*5*); these findings are in accordance with the 70% to 73% resistance rates we observed in isolates from Spain. Similarly, the 77% to 79% rates of ciprofloxacin resistance among our isolates from Thailand concur with the surveillance data, indicating that fluoroquinolone resistance rates already exceed 80% in Thailand (*4*).

Our study provides data on *C. jejuni* fluoroquinolone resistance in 40 countries and on four continents, rendering possible the evaluation of the relative risk for a Finnish traveler to acquire an infection by ciprofloxacin-resistant *C. jejuni* in different travel destinations. When assessing the actual infection rate by ciprofloxacin-resistant *C. jejuni* in any destination, the number of ciprofloxacin-resistant *C. jejuni* isolates imported from that destination during the study months should be divided by the number of simultaneous trips from Finland. In our study, the total number of resistant *C. jejuni* isolates imported to Finland was not known, since we examined isolates from one hospital only. Thus, when the numbers of ciprofloxacin-resistant *C. jejuni* isolates identified were divided by the simultaneous numbers of trips from the whole country, the figures (referred to as speculative infection rates in Table 2) did not provide any real data on infection rates by resistant isolates. Nevertheless, these figures could be used to calculate rate ratios between different travel destinations. Despite the high proportions of ciprofloxacin-resistant isolates in Spain (71%) and Portugal (64%), the risk of acquiring fluoroquinolone-resistant campylobacteriosis appeared to be 10 times smaller in those countries than in Thailand. These results are in line with our previous results, which showed that a tourist’s risk of acquiring quinolone-resistant salmonellosis was significantly higher in Thailand and Malaysia than in other travel destinations (*11*).

In conclusion, we demonstrated a significant increase in ciprofloxacin resistance among all *C. jejuni* travelers’ isolates, as well as among the isolates from Asia alone. The rate of ciprofloxacin resistance remained on a high level throughout the study in Spain and Thailand, the two most frequent countries of origin of the ciprofloxacin-resistant isolates. These data support the concept of continuous selection pressure for the emergence and spread of fluoroquinolone resistance not only in Asia but also in many other parts of the world. Efforts should be made to elucidate and alleviate the factors behind this selection pressure.
